# Proteomic Advances in Cereal and Vegetable Crops

**DOI:** 10.3390/molecules26164924

**Published:** 2021-08-14

**Authors:** Rubén Agregán, Noemí Echegaray, María López-Pedrouso, Rana Muhammad Aadil, Christophe Hano, Daniel Franco, José M. Lorenzo

**Affiliations:** 1 Centro Tecnológico de la Carne de Galicia, Adva. Galicia n° 4, Parque Tecnológico de Galicia, San Cibrao das Viñas, 32900 Ourense, Spain; rubenagregan@ceteca.net (R.A.); noemiechegaray@cetec.net (N.E.); 2 Department of Zoology, Genetics and Physical Anthropology, University of Santiago de Compostela, 15872 Santiago de Compostela, Spain; mariadolores.lopez@usc.es; 3 National Institute of Food Science and Technology, University of Agriculture, Faisalabad 38000, Pakistan; dilrana89@gmail.com; 4 Laboratoire de Biologie des Ligneux et des Grandes Cultures, INRA USC1328, Orleans University, CEDEX 2, 45067 Orléans, France; hano@univ-orleans.fr; 5 Bioactifs et Cosmétiques, CNRS GDR 3711 Orléans, CEDEX 2, 45067 Orléans, France; 6 Área de Tecnología de los Alimentos, Facultad de Ciencias de Ourense, Universidad de Vigo, 32004 Ourense, Spain

**Keywords:** plant proteomics, bottom-up approach, top-down approach, cereal, vegetable crops, food safety, food quality, plant diseases

## Abstract

The importance of vegetables in human nutrition, such as cereals, which in many cases represent the main source of daily energy for humans, added to the impact that the incessant increase in demographic pressure has on the demand for these plant foods, entails the search for new technologies that can alleviate this pressure on markets while reducing the carbon footprint of related activities. Plant proteomics arises as a response to these problems, and through research and the application of new technologies, it attempts to enhance areas of food science that are fundamental for the optimization of processes. This review aims to present the different approaches and tools of proteomics in the investigation of new methods for the development of vegetable crops. In the last two decades, different studies in the control of the quality of crops have reported very interesting results that can help us to verify parameters as important as food safety, the authenticity of the products, or the increase in the yield by early detection of diseases. A strategic plan that encourages the incorporation of these new methods into the industry will be essential to promote the use of proteomics and all the advantages it offers in the optimization of processes and the solution of problems.

## 1. Introduction

Vegetables have been used as a primary resource in the human diet since ancient times. The cultivation of cereals and other vegetables improve and diversify the diet by being an important source of nutrients, vitamins, secondary metabolites, and other compounds that contribute to health and nutrition [1]. Cereals, such as wheat, maize, or rice are consumed all over the world and represent an essential source of carbohydrates and nutrients for the diet [2,3,4]. Cereals are also a fundamental part of the livestock diet, including maize and other coarse grains, such as sorghum, barley, or oats [5], and, together with those used in human nutrition, represent a powerful economic engine for many countries and the livelihood of their families. Other vegetable crops, such as lettuce, zucchini, tomato, eggplant, cucumber, onion, peas, or potatoes are widely consumed as an important source of starch and cellulose, which also contain a wide variety of vitamins and minerals [6].

The cultivation of plants for human consumption has become a business that moves millions of dollars around the world and that supplies food to a world in constant demographic expansion. In this context, much of the success lies in ensuring the control of production, quality, and food safety; these are the elementary pillars of the food industry. Proteomics is a tool born in the field of biology focused on understanding the expression of proteins in a biological system, including all the isoforms and modifications present, as well as the interactions between their structures [7]. The study of proteomes is an arduous and complex task that currently cannot be solved by a single and simple approach. However, the technologies developed in the last decades have contributed to considerably improve the speed, precision, and level of detail [8]. In this review, two major proteomic approaches (bottom-up and top-down) based on separation techniques and mass spectrometry (MS) for the resolution and protein characterization are discussed.

The applicability of proteomics in the different fields of human activity is enormous, with the potential to improve processes and increase productivity. In this review, the authors put the spotlight on plant proteomics research and how it is opening the doors to the development of a technology that enables advancement in production control, quality assurance, and improvement of human health from a sustainable perspective. Considering current population growth and the enormous pressure on agricultural production in the coming decades, this model of action, known as translational plant proteomics, has the potential to guarantee a controlled and adequate supply of plant crops through the development and application of new practices committed to the environment, the conservation of biodiversity, and human health [9].

## 2. Role of Vegetable Proteins in Agriculture and Food

Cereal grains constitute a large part of the plant proteins in the human diet. They are considered the main food for the majority of the world’s population [10]. In some less developed countries, the importance of cereal consumption acquires special relevance as the protein intake is distributed among different dietary sources, such as meat, legume seeds, or cereals, protein intake in the diet depends on a single cereal crop [11].

The importance of vegetables for the food industry is given by their complex chemical composition that provides specific benefits in the diet by contributing to body hydration due to their high-water content and the regulation of metabolism, among other positive functions. In general, they are highly nutritious foods with a low caloric load, where the protein content can vary between 0.5% and 1.5%, except for green legumes (5–6% in peas and beans) and dried legumes (20–34% in lentils, dried beans, and soybeans) [6]. In cereal grains, the protein fraction is more abundant. It is the second most important component after carbohydrates with a variable content between 7.50 and 16.68% of the grain [12]. Despite their low protein content, vegetable crops are a good source of amino acids. They contain all the essential amino acids, although unlike other foods such as eggs or milk, the proportions of some of these amino acids are not sufficient for a correct diet. Furthermore, other nonprotein amino acids, such as homoarginine, carnitine, citrulline, taurine, or α-aminobutyric and γ-aminobutyric acids, are also present in plants with a direct impact on consumers’ health. On the other hand, free amino acids play an important role in the acceptance of vegetables by providing flavours such as sweet (glycine and alanine), bitter (valine and leucine), and umami (aspartic acids and glutamate) [13].

In cereal grains, the protein fraction is more abundant. It is the second most important component after carbohydrates, with a variable content between 7.50 and 16.68% of the grain and, according to its solubility in different solvents, it can be classified into albumins, globulins, prolamins, and glutelins [12]. Unfortunately, cereals are not a balanced source of amino acids. They have a slightly deficient content in some essential amino acids, especially lysine, and to a lesser extent tryptophan [14]. Table 1 shows the amino acid profile of some of the most cultivated cereals in the world.

In addition, to serve as an energy source and provide the necessary nutrients for the correct growth and maintenance of the body, plant matrices may serve as a reservoir of bioactive peptides that may act as functional and health-promoting ingredients. These types of compounds have been found in plant foods as diverse as pseudocereals, algae, edible fungi, garlic, turmeric, sesame, peanut, alfalfa, tubers, or spinach. Recently, legumes and cereals are the most intensively investigated crops due to their high distribution in the food market due to their importance as a valuable protein source in the diet [15]. Some peptides present in legumes have been found to help maintain health by exerting different biological activities (e.g., cardiovascular, endocrine, antimicrobial, antimutagenic, immunological, and neurological). These positive effects depend on the amino acid sequence and the similarity between peptides, which have been found to share some common features, such as short residue chains (2 to 10), the presence of hydrophobic residues, the presence of proline, lysine, and arginine, and resistance to proteolysis [16].

Apart from human consumption, cereals play a fundamental role in animal nutrition. 70% of the arable land is dedicated to planting cultivation for animal feed [17]. Some crops, such as corn, wheat, or barley, are widely used and demanded, and account for up to 60% of the energy ingested by livestock [18]. Specifically, corn is produced and used mainly as animal feed. Only 15% of its grain is intended for human consumption. Sorghum production in the United States, the second-largest producer after Nigeria, is largely focused on animal feed [19].

**Table 1:** Amino acid composition of the main cultivated cereals and comparison with the daily requirements of amino acids stipulated by the WHO/FAO/UNU.

AA (mg/g of Grain)	^a^ Wheat	^b^ Rice	^c^ Oat	^d^ Sorghum	^e^ Barley	^f^ Adult AA Requirements	
EAA						AA	Quantity (mg/kg per day)
Histidine	2.38	1.33	3.98	2.30	2.12	Histidine	10
Isoleucine	8.22	1.90	4.76	4.00	3.47	Isoleucine	20
Leucine	10.76	4.71	9.68	13.90	5.79	Leucine	39
Lysine	2.54	2.59	4.36	2.30	3.88	Lysine	30
Methionine	8.70	1.66	1.97	1.70	1.68	Methionine	10
						Methionine + cysteine	15
Phenylalanine	6.13	3.08	6.03	5.30	4.06	Phenylalanine + tyrosine	25
Threonine	3.01	2.25	4.54	3.60	3.29	Threonine	15
Tryptophan	-	-	-	-		Tryptophan	4
Valine	8.15	2.77	6.50	4.80	4.68	Valine	26
∑EAA	49.87	20.29	41.79	37.90	28.97		
NEAA							
Alanine	4.87	3.24	5.87	9.90	4.03		
Arginine	4.38	5.87	10.33	4.20	5.56		
Asparagine	5.54	5.13	9.30	7.40	5.65		
Cysteine	8.38	-	-	2.10	1.94	Cysteine	4
Glutamine	29.33	9.30	26.94	22.60	19.65		
Glycine	4.44	2.87	6.23	3.30	4.15		
Proline	10.41	2.63	7.63	8.50	8.79		
Serine	4.56	3.04	5.84	5.10	4.03		
Tyrosine	4.76	1.70	3.98	4.30	2.94		
∑NEAA	76.65	33.77	76.10	67.40	56.74		

^a^ Jiang et al. [20]; ^b^ Sekhar and Reddy [21]; ^c^ Sterna et al. [22]; ^d^ Waggle et al. [23]; ^e^ Smith [24]; ^f^ Millward [25]; WHO/FAO/UNU: World Health Organization/Food and Agriculture Organization/United Nations University); AA: amino acid; EAA: essential amino acid; and NEAA: non-essential amino acid.

## 3. The Study of the Proteome. Technologies and Techniques

**Figure 1:**
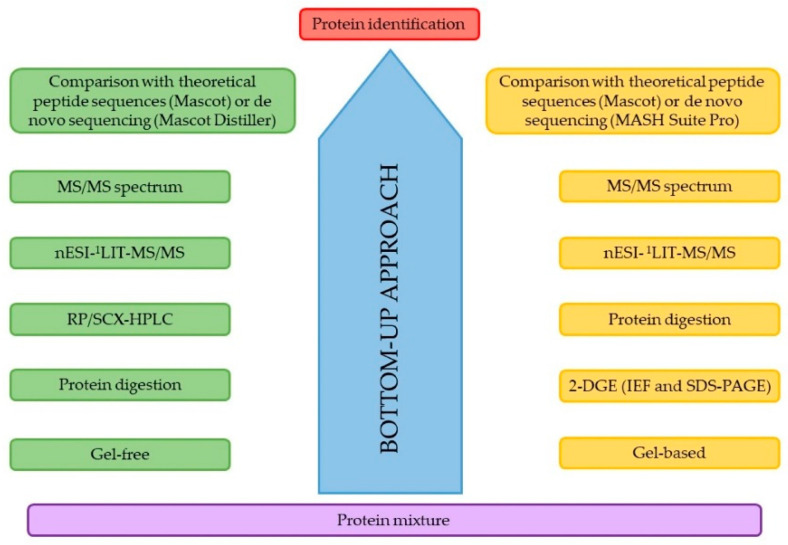
Typical workflow of a proteomic assay following a bottom-up and top-down strategy. ^1^ LIT: linera ion trap.

Since the completion of the human genome project at the beginning of this century, efforts by the international scientific community have focused on deciphering such genome and mapping the resulting protein expression [26]. In summary, the expression of the genome leads to the formation of the transcriptome, which is defined as the complement of mRNAs that reflects all the expressed genes of an organism, and their subsequent conversion to the respective protein products forms the corresponding proteome [27]. This term first appeared in the early ‘90s when Marc Wilkins and collaborators suggested a defining word which encompasses those PROTEins expressed by the genOME, and was publicly presented during the first proteomics conference in the Italian city of Siena in 1993 [28]. Therefore, the proteome can be defined as the set of proteins encoded by the genome of any living being, which includes all the isoforms and modifications present, as well as the interactions between them, their structures, and higher-order complexes [29]. For the study of this specific set of proteins, the area encompassed within biology known as proteomics was born, formed by the words “protein” and “genomics”, first coined in the mid-90s, although the concept was previously proposed by Norman G. Ander and N. Leigh in 1979 when they tried to unlock the human genome by identifying the respective proteins using the 2DE separation technique in the presentation entitled “Human Proteins Index Project” [30]. Today, MS technology is indispensable for the study of the proteome, which is combined with a wide variety of separation methods in protocols involving enzymatic digestions of the protein mixtures prior to spectrometric analysis. This analytical strategy is known as the bottom-up approach and is still the main strategy used in proteomics. Figure 1 briefly depicts a flow diagram with two summary examples of classical protocols for the bottom-up approach.

In the last decades, protein mixture separation protocols included gel electrophoresis and liquid chromatography (LC). The two-dimensional gel electrophoresis (2-DGE) allows the separation of complex samples with a single run. This is a fundamental characteristic that has allowed the analysis of hundreds of proteins in the same gel, which has led to an increase in its popularity as a separation technique. Furthermore, other aspects associated with this technique have contributed to its use in proteomics, such as the simplicity of equipment, the delivery of reliable and reproducible results, and the compatibility with analytical techniques such as MS [31]. Many times, the spots in the electrophoretic gel can contain more than one protein, as the resolution of the technique is not perfect. The excision of the spot and its analysis by MS enables the identification of the individual proteins that compose it [32]. Before the MS analysis, the spot is digested by proteolytic enzymes (endoproteases/endopeptidases). This step is essential in bottom-up approach and differential with the top-down approach and allows us to increase the comprehensiveness of the proteomic analysis. Trypsin is the most frequently used protease due to the production of peptides suitable for MS analysis, its high specificity, and reasonable cost, covering 96% of the studies under a bottom-up approach. The second most used protease is Lys-C, followed by chymotrypsin, Glu-C, and pepsin [33]. On the other hand, the production of short peptides with Arg or Lys at the C-terminus as result of the trypsin digestion favored their separation by modern chromatographic methods, fragmentation, and identification based on search algorithms [34]. However, the small size of the trypsinized proteins, between 5 and 20 amino acids, makes it difficult to cover the entire proteome [35]. The subsequent ionization of peptides is carried out by techniques such as Matrix-assisted laser desorption/ionization (MALDI) and electrospray ionization (ESI). MALDI is partially resistant to interferences caused by buffers commonly used in proteomics (e.g., tris or urea), and thus the formation of singly charged ions is promoted, facilitating the interpretation of mass spectra. On the contrary, ESI promotes the generation of multiply charged ions [36]. Different techniques are used in the breakdown of peptides ions. The dissociation treatment can be performed using collision-induced dissociation (CID), electron-capture dissociation (ECD), or electron-transfer dissociation (ETD) [35].

Liquid phase separation by LC or capillary electrophoresis (CE) provides an alternative to 2-DGE separation and offers some advantages over it, such as higher sensitivity, higher dynamic range, ease of automation, and speed. In addition, the different separation mechanisms, such as size exclusion, reversed-phase (RP), or ion exchange, allow the analysis of highly acidic or basic proteins of any molecular weight [37]. The one-dimensional separation LC using the RP column is the most widely used mechanism for peptide resolution. On the other hand, when multidimensional separation is used, RP and strong cation exchange (SCX) columns are the preferred combination [38]. This combination of mechanisms improves the resolution of the sample before its analysis by MS. In the last step of the spectrometric analysis, the generation of mass spectra can be carried out using various available mass analyzers. The mass analyzers used in proteomics are quadrupole (Q), ion trap (IT), time-of-flight (TOF), Fourier transform ion cyclotron resonance (FTICR) and orbitrap, with different parameters such as sensitivity, resolution, or mass accuracy. In addition, they can be put together in tandem (MS/MS) taking advantage of a different physical property of the ion motion for mass-to-charge based separation [36].

The combination of LC with MS/MS technique (LC-MS/MS) after enzymatic digestion of proteins is a high-throughput strategy currently widely used for the proteomic analysis of complex samples [39]. This new proteomic analysis strategy, called shotgun proteomics, is gradually replacing 2-DGE proteomics by significantly increasing the sensitivity of mass spectrometers [40]. Advantages and disadvantages of these two different proteomic methods are summarized in Table 2. Using shotgun proteomics, hundreds of thousands of peptide species could be resolved in few hours [41]. For the relative quantitative analysis of proteins in the shotgun approach, label and label-free quantification (LFQ) methods are available [42]. In the first group of methods, the modification of samples with molecules containing isotopes can be performed by methods, such as stable isotope labeling with amino acids in cell culture (SILAC), stable isotope labeling in mammals (SILAM), isotope affinity tagging (ICAT), dimethyl labeling, and isobaric tags for relative absolute quantification (iTRAQ) and tandem mass tag (TMT). In label-free quantification, methods such as spectral count and ion intensity/AUC are used [41].

A new proteomic strategy called targeted proteomics, based on the approach towards the detection of specific proteins, offers the advantage of working with a limited portion of the proteome instead of quantifying thousands of proteins without any prior knowledge [43]. This approach differs from shotgun in that, instead of finding candidate biomarkers to use in the detection of an event, such as a plant disease, a pre-established biomarker is already used for the positive detection of such event. That is, targeted proteomics focuses on the quantification of one or a set of proteins of interest rather than profiling samples and comparing them to find the differential protein abundance in an unbiased manner. In this way, these protein biomarkers can be measured in a large set of samples with high precision and reproducibility [44]. Three groups of targeted proteomic approaches are distinguished: selected reaction monitoring (SRM), also known as multiple reaction monitoring (MRM), parallel reaction monitoring (PRM), and data-independent acquisition (DIA) combined with targeted data extraction of the MS/MS spectra [45].

After the spectrometric analysis, different search tools are available for the interpretation of the mass spectra. Some of the most used are software such as PLGS (ProteinLynx Global server), Mascot, developed by the company Matrix Science, or PEAKS. However, de novo sequencing of raw MS/MS spectral data is sometimes used to determine novel proteins, mutations, and post-translational modifications (PTMs). This approach requires acquiring the data and determining mass and composition of the peptides directly from the MS/MS spectrum and predicted fragmentation. Some of the most used logarithms in de novo sequencing method are PEAKS, PepNovo, Mascot Distiller, or UniNovo [35].

## 4. Benefits of Proteomics in the Production of Cereals and Vegetable Crops

### 4.1. Concept of Translational Plant Proteomics

The knowledge generated by the basic sciences has always served as a fundamental support to solve problems related to human activity. “Translation” is a word derived from the Latin word “translatio”, which means to bring. This transfer of knowledge from the academic research field to another application is not new to the medical field. The term “translational research” is commonly used in medicine for the process of transferring scientific discoveries to clinical applications in the struggle against cancer. Therefore, “translational proteomics” can be defined as the emerging sub-discipline within the field of proteomics in charge of translating the knowledge generated by basic science for the resolution of problems related to human activity. Thus, translational plant proteomics is focused on activities related to the recreational and economic values of plants, food security, and energy sustainability [46].

The following sections of this review discuss proteomics applications in plant foods that cover such important areas of food technology as safety assessment, quality and authenticity, and process optimization and monitoring.

### 4.2. Safety Assessment

#### 4.2.1. Detection of Allergens

The plant tissues that we consume are composed of thousands of different proteins, and some of these may cause an allergic response in atopic individuals, causing health problems that can even have a fatal outcome. The allergenicity of these proteins will depend on a series of factors such as their belonging to a specific family, their abundance, and their stability to processing and digestion [47]. Fortunately, most allergens in plants belong to a few families and superfamilies of proteins, such as the cupin superfamily, found in seeds, or the prolamine superfamily. Some examples of widespread and important allergies caused by plant foods are those that occur as a result of the consumption of storage proteins found in products such as peanuts and cereals [48]. Along with fruits, fresh vegetables are the main source of vitamins and minerals in the human diet. Vegetables, such as tomatoes, lettuce, asparagus, cabbage, celery, fennel, carrots, and artichokes, contain significant levels of allergens. In addition, food allergies have become widespread around the world in recent decades, presumably due to factors including the composition of food products, diversification of food sources, and environmental practices [49]. Therefore, the detection of possible allergens from foods of plant origin, especially cereals, is a primary issue for the food industry. In this sense, wheat allergy is one of the most widespread food allergies. The allergens involved in the immune responses to their consumption were identified as proteins mainly associated with gluten, in particular ω-5 gliadins and high-molecular-weight glutenin subunits [50].

A proteomic approach could be applicable in the detection of food allergens. Conventional 2-DGE analysis, followed by Western immuno-blotting with sera from allergic patients, and MS analysis have been shown to be effective to this purpose [51]. Akagawa et al. [52] identified the presence of 18 allergens in wheat flour using a gel-based proteomic approach. The proteins were separated by 2-DGE using IEF and SDS reaching a high resolution. IgE-binding proteins were detected by immunoblotting with sera of patients with a food allergy to wheat, and after digestion with trypsin, the peptides of IgE-binding proteins were analyzed by MALDI-TOF MS/MS, identifying nine subunits of low molecular weight glutenins as the proteins that showed a greater binding to IgE. Traditionally, immunoassays, such as the enzyme-linked immunosorbent assay (ELISA), have been widely used to quantitatively determine the presence of a particular known antigen in the detection of food allergens. However, in the case of ELISA, a series of disadvantages associated with protein denaturation and epitope degradation during the thermal process led to its gradual replacing by targeted MS/MS techniques, which represents an extraordinary improvement in monitoring a specific set of proteins using SRM by quadrupole-orbitrap (Q-orbitrap) mass analyzer [53]. LC-MS/MS-MRM system was successfully used in the detection of allergens in sesame seeds by identifying specific biomarkers [54]. In the same vein, Boo et al. [55] used the same approach in the detection and quantification of peanut allergens in sugar cookies. The method provided good recovery of allergen marker peptides in this food (5–500 ppm), offering efficiency, sturdiness, and affordable costs for use as a routine assay.

One of the most universal diet-related adverse reactions in nutrition is gluten intolerance. This compound is the main storage protein in wheat grain, and comprises a mixture of hundreds of proteins, mainly gliadin and glutenin, the intake of which causes the well-known celiac disease that affects to 1% of the western population [56]. Importantly, celiac disease is different from wheat allergy. In the former, a specific protein (gluten) triggers a different type of abnormal immune system reaction. These immune problems are considered of special relevance due to the high consumption of wheat in countless prepared foods such as pasta, pastry products (cookies, muffins, or cakes), and dairy and meat products. Fiedler et al. [57] demonstrated the feasibility of a targeted proteomic approach based on the SRM method for the detection of gluten contamination in oatmeal. The peptides, resulting from extracting prolamines from wheat, barley, rye, and oat flours with chymotrypsin, were subjected to LC-MS/MS. Using this protocol, two wheat peptide markers were detected from the α/β-gliadin protein at a concentration of 10 ppm of wheat flour in oatmeal. By comprising gluten around 10% of the protein in wheat flour, it is possible to detect up to 1 ppm of wheat gluten in oatmeal. In the same vein, Martínez-Esteso et al. [58] applied an nLC-MS/MS method based on SRM approach to find specific peptides from wheat gluten. The authors subjected gluten to multi enzymatic digestion with lysC, trypsin, and chymotrypsin after fractionation using RP-HPLC. Subsequently, a separation of the peptides and spectrometric analysis using ultra-performance liquid chromatography (UPLC) and TOF-MS, respectively, identified 434 peptide sequences that were subsequently screened by UPLC-ESI-MS/MS based on SRM according to the presence of immunogenic and toxic sequences for those allergic individuals.

Proteomics can be a useful tool in the study of the presence of allergens in genetically modified organisms. Unwanted changes in protein expression from transgenic crops may harm consumers’ health. Therefore, an evaluation of novel foods is imperative. García-Molina et al. [59] successfully performed a trial with a proteomic approach based on 2-DGE, followed by RP-HPLC/nESI-MS/MS for the comparison of two genetically modified wheat lines, focusing on a lower expression of gliadin, responsible for allergies, and unprocessed wild wheat for reference. Allergic reactions in the plant kingdom are not exclusive to gluten-containing seeds. Soybean is responsible for frequent allergic reactions and is one of the most commercialized genetically modified crops. An approach using a 2-DGE, followed by Western blotting with the plasma of plant-sensitive individuals and an MS/MS analysis for the identification of protein-IgE bindings, could be an adequate strategy for the evaluation of food safety in soybeans and other transgenic vegetable crops [60,61]. In general terms, hardly any new allergens have been identified in genetically modified organisms using comparative proteomic techniques. In most studies, the protein profiles showed little variation between the genetically modified organism and the natural control. It should be noted that many proteome modifications are related to conventional reproduction and genotypic variation [62].

#### 4.2.2. Detection of Pathogenic Microorganisms

Pathogenic microorganisms can reach plants during the production chain from various sources. Pathogen populations are capable of establishing themselves on crops during growth, often from contaminated irrigation water or contaminated surface runoff water from nearby grasslands. The application of raw or improperly composted animal remains to the soil, contaminated fertilizers, the presence of sewage and insects are other possible causes of contamination during the pre-harvest stage. Once the plant has been harvested, poor handling and/or poor processing of plant material may favour the presence and proliferation of pathogens, such as unintentional cuts or the use of non-potable water [63]. Coliform bacteria are often found in vegetables due to faecal contamination. Several pathogens belonging to this group, such as *Enterobacter cloacae* and *Klebsiella* sp. have been isolated from lettuce [64]. Other pathogens typically associated with vegetable crops are Salmonella and Shigella, also isolated from lettuce and other crops, such as tomato, cantaloupe, mamey, and scallions and parsley, respectively [65]. The microorganism genera *Salmonella*, *Escherichia*, especially *Escherichia coli* O157: H7 and *Shigella*, generate special interest due to their frequent involvement in diseases transmitted by the consumption of contaminated plant foods [66], responsible for numerous outbreaks around the world. These pathogens release a series of toxins of a protein nature (e.g., enterotoxins, neurotoxins, leukocidins, and hemolysins) as a result of their metabolism, undetectable for consumers as they do not generate strange odours, flavours, or appearances, which serve to favour their proliferation in the plant tissues [67].

Proteomic techniques may contribute to the early detection of these microorganisms in agricultural foodstuffs, improving the comprehension of the infection mechanisms, antibiotic resistance, and biofilm formation [68]. This early detection is usually carried out by identification specific proteins that function as biomarkers, revealing the presence of a particular microorganism within a sample. On the other hand, these “fingerprints” can be used to differentiate some microorganisms from others, with a sufficient level of detail to classify them by species [69]. These biomarkers could become essential to guarantee the safety and authenticity of foods, ensuring rapid action in a possible outbreak event. A fast and effective protocol is imperative in the current market where products travel long distances [70]. MALDI-TOF-MS is a fast and effective technique for the identification of foodborne pathogens. The result of this analysis technique is a characteristic spectrum or fingerprint, where the main proteins used for identification are ribosomal proteins, which represent between 60 and 70% of the dry weight of a microbial cell [71]. This methodology has been successfully applied in the identification of spoilage microorganisms in plant foods. Six biomarker proteins of *Escherichia coli* O157: H7, responsible for numerous outbreaks in plant foods [72,73,74], were identified using this approach [75]. Fagerquist et al. [76] demonstrated the speed and high specificity of the MALDI-TOF/TOF MS/MS technique for the identification of Shiga toxin 2 subtypes from Shiga toxin-producing *Escherichia coli* using MALDI-TOF/TOF MS/MS. In another study, an identification confidence score at a concentration of 100 and 1000 cfu/mL higher than 85% was achieved for four pathogens (*Escherichia coli*, *Salmonella typhimorium*, *Staphylococcus aureus*, and *Listeria monocytogenes*) recovered from various classes of plants, proving to be a time-effective technique for the identification of pathogens in foods [77].

The MALDI ion source is especially indicated for those studies that involve proteins as it allows the analysis of large molecules without causing their fragmentation. A protocol for finding biomarkers based on MALDI-TOF-MS increases the speed and dynamism concerning classic bacterial identification protocols, such as phenotypic tests, classical culture, or immunological tests, achieving a high specificity in the identification of species (around 95%). However, the steps before this protocol are time-consuming, as the isolation of the bacterial strains is required, and the analysis of mixed cultures consisting of many different strains is not yet possible, as the number of overlapping signal ions increases considerably [78]. These limitations can be overcome by replacing the MALDI ion source with an ESI. In this way, a higher mass resolution can be achieved. On the other hand, the combination with separation techniques such as LC provides a more precise and efficient identification of biomolecules in complex mixtures and matrices, allowing the discrimination of bacteria down to the strain level [78]. In a comprehensive study, Calo-Mata et al. [79] used LC-ESI-MS/MS with a top-down approach for the description of unique markers in the identification of bacterial pathogens. The tryptic digestion of proteins extracted from *Streptococcus* spp., *Enterococcus* spp., *Bacillus* spp., *Listeria* spp., *Salmonella* spp., *Enterobacteriaceae*, and *Pseudomonas* spp. provided information for the identification of potential markers for discrimination between species of bacteria. The study demonstrated the aptitude of cysteine synthase and cysteine proteases stophapain A and stophapain B and 1-pyrroline-5-carboxylate dehydrogenase proteins to be used in Staphylococcus aureus differentiation. The peptides resulting from the use of cysteine protease were specific for this bacterium and could be used for the rapid and direct detection of pathogens in food products.

Fungal contamination of crops is another serious biohazard problem. Of particular concern is the presence of mycotoxins (e.g., e aflatoxins, ochratoxins, and trichothecenes) in cereals and animal feed. These toxins are secondary metabolites produced by various types of fungi, mainly those belonging to the genera *Aspergillus*, *Penicillium*, and *Fusarium*, which enter the food chain through contaminated crops. Their intake has been linked to dangerous events for health, such as mutagenesis and gastrointestinal and renal failure [68]. Therefore, the presence of fungi in crops can be dangerous and proteomics, as in the case of bacteria, can help identify their presence. Wigmann et al. [80] applied MALDI-TOF-MS to distinguish members belonging to the fungus species *Fusarium fujikuroi*, some of which are producers of mycotoxins. The target was to differentiate closely related microorganisms and to validate the efficacy of a standardized protocol by identifying field isolates that meet the morphological characteristics of the species. The authors created a database and demonstrated its discriminative power with 94.61% correct identifications to the species level. Similarly, Quéro et al. [81] developed a database for the correct identification of filamentous fungi responsible for the decomposition of food, which are frequently involved in food spoilage and cause significant losses and substantial economic damage. MALDI-TOF-MS has once again become a key tool in the differentiation of fungal species, specifically filamentous fungi. After applying a standardized extraction protocol, the authors acquired 6477 spectra of 618 strains of fungi belonging to 136 species. The performance of the database achieved was then evaluated by cross-validation, achieving approximately 95% correct identification to the species level. The database was also contrasted with external isolates belonging to 52 species, being able to identify 90% of the fungi to the species level.

### 4.3. Authenticity and Quality Assessment

Today, there is strong control over the labelling of food products, which are governed by strict laws. Even so, due to the growing demand for food and trade globalization, frauds in the nutritional declaration of certain products continue to occur, either deliberately or due to unintended errors throughout the food chain. An example of common fraud is substituting key ingredients for similar, lower quality, and cheaper ingredients [82]. The adulteration of food implies the incorporation of useless, harmful, and unnecessary substances that diminish its quality and that can contribute to deplete the health of consumers by depriving them of the necessary nutrients to maintain adequate health or cause poisoning and allergic problems due to non-declaration of certain compounds [83]. A frequent type of fraud in the cereal industry, specifically in the flour manufacturing industry, is the partial substitution of durum wheat (*Triticum turgidum* ssp. *Durum* L.) destined mainly for the production of pasta due to its rheological characteristics with the cheapest common wheat (*Triticum aestivum* L.) used in baking operations. To detect adulteration of this calibre, an analysis strategy based on LC-MS/MS, together with proteolytic digestion may offer adequate selectivity and sensitivity. Russo et al. [84] found this approach feasible for the analysis of adulterations in samples of durum wheat. Applying UPLC-ESI-MS/MS based on MRM for selective and sensitive detection of common wheat in samples of durum wheat they found a new species-specific peptide marker derived from the tryptic digestion of the Pin-a protein selectively present in common wheat. Therefore, it might be an adequate method for the routine detection of adulterations along the durum wheat production chain, detecting the presence of common wheat at a concentration as low as 0.01% (*w*/*w*). The targeted analysis allowed the identification of unique marker peptides for each cereal, even those closely related [85]. Using the same approach, Bönick et al. [86] described a correctly applied biomarker peptide analysis strategy for the differentiation of common wheat, spelt, and rye. The specific peptides for wheat, spelt, and rye were used satisfactorily in the verification of the presence of these three types of flours in bakery products at a level of 0.5–1%. The higher the number of specific peptides identified, the more reliable the differentiation among species will be, especially those closely related. In this sense, a strategy directed towards obtaining more appropriate enzymes could facilitate this task by applying bottom-up proteomics approaches.

More and more countries are adopting regulations regarding the labelling of genetically modified organisms. For example, the EU regulation requires the correct labelling of food and feed that contains a threshold of 0.9% for transgenic ingredients [87]. Methodologies based on proteomic approaches can help to control the quality of these organisms, essential in the prevention of risks inherent to their consumption, as production of genetically modified organisms could generate new proteins or the overproduction of existing ones with a negative impact on consumers’ health, as previously commented concerning the presence of allergens. It is important to keep in mind that any transgenic food must be considered as safe as its natural counterpart as long as it has the same characteristics and composition [88]. Differences in the expression of proteins between the genetically modified organism and its natural counterpart can be established utilizing 2-DGE techniques. However, different intrinsic problems associated with electrophoretic systems have been reported, such as different sample preparation methodologies, uncontrollable variations inherent to biological systems, the not-complete reproducibility of gels, the tediousness of the method, and the possible presence of more than one protein in the same spot [89]. The coupling of gel-based methods with MS in the study of transgenic plant foods has provided more robust protein profiles for the establishment of comparisons with conventional products. Numerous studies with this approach have been proposed to assess the quality of different genetically modified plant products (Table 3).

The application of iTRAQ quantitave proteomics for the identification of differentially expressed proteins in GMOs has been reported to be an effective method. The use of a shotgun approach instead of methods that involve the use of 2-DGE represents a qualitative step forward, leaving behind problems such as weak ability to detect low-abundance proteins, narrow linear dynamic range of detection, copious simple handling, and low reproducibility [90]. Liu et al. [91] used shotgun proteomics with the iTRAQ as a labelling method for the evaluation of differences in the proteomic profile in seed cotyledons from four genetically modified and three natural genotypic soybean lines. In total, the authors found 170 differentially expressed proteins in the three transgenic soybean lines with the same parents, while 232 were identified in the three natural soybean lines. Therefore, the exogenous introduction of genes was harmless, causing fewer variations in protein expression than those found in their natural counterparts. A similar result was found in maize after applying iTRAQ quantitative proteomics. The insertion of foreign genes hardly modified the protein profile, causing changes only in abundance and ruling out the appearance of new unintended proteins, toxins, or allergens [92].

**Table 3:** Proteomic approaches for quality assessment in different genetically modified plant foods.

Food	Purpose of Analysis	Target	Proteomic Techniques	Reference
Beans	Comparison between transgenic (Embrapa 5.1) and natural bean	Grain proteome	2-DGE: IEF and SDS-PAGE	Balsamo et al. [93]
			MS: MALDI-TOF MS and MALDI-TOF MS/MS	
Maize	Comparison between transgenic (MON810) and natural maize	Seed proteome	2-DGE: IEF and SDS-PAGE	Zolla et al. [94]
			MS: ^a^ nHPLC-MS/MS	
Maize	Comparison between transgenic (MON810) and natural maize	Flour proteome	2-DGE: IEF, SDS-PAGE, and 2D-DIGE	Vidal et al. [95]
			MS: ^b^ nUPLC-^c^ nESI-^d^ QTOF-MS/MS	
Maize	Comparison between phytase transgenic and natural maize	Seed proteome	2-DGE: IEF and SDS-PAGE	Tan et al. [96]
			MS: MALDI-TOF MS/MS, iTRAQ, and ^e^ nLC-MS/MS	
Potato	Comparison between transgenic (S/CDI-expressing lines) and natural potato	Tuber proteome	2-DGE: IEF and SDS-PAGE	Khalf et al. [97]
			MS: LC-ESI-MS/MS	
Rice	Comparison between transgenic (Bar68-1 and 2036-1a) and natural rice	Seed proteome	2-DGE: 2D-DIGE	Gong et al. [98]
			MS: MALDI-TOF MS/MS	
Rice	Comparison between transgenic (Bt and PEPC) and natural rice	Seed proteome	2-DGE: IEF and SDS-PAGE	Xue et al. [99]
			MS: MALDI-TOF MS/MS	
Soybean	Comparison between transgenic (MSOY 7575 RR) and natural soybean	Seed proteome	2-DGE: IEF and SDS-PAGE	Brandão et al. [89]
			MS: MALDI-^d^ QTOF MS	
Soybean	Comparison between transgenic (MSOY 7575 RR) and natural soybean	Seed proteome	2-DGE: IEF, SDS-PAGE, and 2D-DIGE	Barbosa et al. [100]
			MS: MALDI-^d^ QTOF MS/MS and ^b^ nUPLC-^c^ nESI-^d^ QTOF MS/MS	
Tomato	Comparison between transgenic (TFM7) and natural tomato	Fruit proteome	2-DGE: IEF and SDS-PAGE	Mora et al. [101]
			MS: ^e^ nLC-ESI MS/MS	

^a^ nHPLC: nanoflow high performance liquid chromatography; ^b^ nUPLC: nano-ultraperformace liquid chromatography; ^c^ nESI: nano-electrospray ionization liquid chromatography; ^d^ QTOF: quadrupole time-of-flight; and ^e^ nLC: nano-liquid chromatography.

### 4.4. Early Detection of Diseases

Pathogens, such as fungi, bacteria, viruses, nematodes, parasitic plants, and pests can attack plants during their development, causing large economic losses. To combat the diseases produced by these biotic agents, a series of modifications are produced at the physiological, biochemical, cellular, and molecular levels in plants, which entail the creation of compounds to reinforce the walls of the cells that surround the infection and create a barrier that prevents the expansion of the infection. On the other hand, the plant can protect itself through the restriction of the growth and expansion of the pathogen throughout the entire plant; even the plant can develop an acquired systemic resistance that gives it the protection that can last from several months to the full season [102]. Therefore, changes occurred in the expression of certain plant proteins which can be studied with currently available proteomic technology. In this way, it is possible to identify the proteins responsible for the pathogenicity and virulence of infectious organisms, revealing the cause of the metabolic changes produced in the plant. Discerning the mechanisms involved in infection could be useful in disease diagnosis and crop protection [103]. Wu et al. [104] found significant differences in the expression of 93 proteins by two-dimensional differential gel electrophoresis (2D-DIGE) between healthy and inoculated sugarcane with mosaic virus. Among these proteins, 17 were not identified before infection. Most of the proteins found were in the chloroplast and cytoplasm of plant cells and were mainly involved in energy and metabolism, stress and defence responses, photosynthesis, and carbon fixation. In another study, Barnabas et al. [105] investigated the modification of the proteome in the meristem of a susceptible sugarcane cultivar infected with the fungus *Sporisorium scitamineum*. Differentially abundant proteins were identified by 2-DGE coupled with MALDI-TOF/TOF-MS. A total of 53 of these differentially expressed proteins were found to play a role in defence, stress, metabolism, protein folding, energy, and cell division in the plant. A non-gel based proteomic approach was carried out by Lin et al. [106] in the study of sweet potato infection by the fungus genus *Fusarium*. A comparative quantitative proteomic analysis was conducted to investigate the defence mechanisms involved in the vegetable using TMT labelling and LC-MS/MS. The authors obtained valuable data on the type of proteins involved in plant defence, such as those associated with signalling transduction, plant resistance, chitinase, and subtilisin-like protease. Indeed, the application of non-gel-based proteomics using LC-MS with a shotgun approach has provided interesting and profitable results in the fight against stress produced by biotic agents. Parker et al. [107] identified 2369 proteins in tomato leaves after infection with *Pseudomonas syringae* pv. *Tomato* DC3000 using iTRAQ LC-MS/MS quantitative proteomics, of which 477 were related to such infection. The subsequent analysis of these differential proteins provided valuable information on the modifications in the protein profile induced by the inoculation of the pathogen and the roles played by these proteins in the defence of the host, extrapolated to crops other than tomato infected by other pathogens. In this comparative proteomic study, multidimensional LC was used for better protein resolution, also compensating for other limitations inherent to two-dimensional gels [108]. The same approach was carried out by [109] for the study of the response of host protein expression involved in tobacco mosaic virus. These authors revealed differential change in protein abundance, involving pathways in protein translation, protein processing, photosynthesis, and plant defence. ITRAQ-based proteomics turned out to be again a useful methodology in deciphering plantar defence mechanisms in response to infections produced by microorganisms. The physiological response of sugarcane to the dreaded red stripe disease by *Acidovorax avenae* subsp. *Avenae* caused the differential expression of 1027 proteins in a resistant cultivar and 1130 proteins in another susceptible cultivar. Most of these proteins were involved in the metabolic, single organisms metabolic, and oxidation-reduction processes, and in catalytic activity [110]. Understanding the changes that occur in the proteomes of resistant and disease-susceptible varieties could be very useful in the molecular improvement of disease resistance.

## 5. Conclusions

Vegetable crops represent an essential pillar in human nutrition, being an important source of starch, fibre, vitamins, minerals, and amino acids. In addition, large areas of land are used for the cultivation of cereals for animal feed. On the other hand, the presence of certain bioactive peptides in vegetables has been associated with a favourable state of health. For these reasons, the use of plant resources has become a priority given the incessant increase in demand. Proteomics opens the door to a series of new technologies that can be applied in the development of vegetable crops through the application of new techniques that could have a favourable impact on aspects as important as food safety, through rapid and reliable detection of the presence of allergens or pathogenic microorganisms, the analysis and guarantee of product quality and authenticity, or the early detection of crop diseases associated in many cases to fungi, which cause considerable economic losses for the producers. Considering the current rate of research and development of proteomics in plants, the promotion of knowledge transfer from the laboratory to companies will be essential in the coming years, favouring the creation of more reliable and faster methods of action that would increase the dynamism of this sector of the food industry. In addition, new job positions could be created for qualified personnel with the capacity to create information and knowledge exchange networks that would favour the continuous improvement of proteomic methods.

## Figures and Tables

**Table 2:** Strengths and weaknesses of 2-DGE-based and shotgun proteomics. Table from Marcus et al. [40].

	2-DGE-Based Proteomics	Shotgun Proteomics
Sample consuming	++(+) *	+
Time consuming	+++	++
Analysis depth	++	+++
Separation/identification		
Separation/detection of proteoforms		
Identification on protein level	Multiple identifications	Only by inference from peptides
Detection of proteoforms	+++	-
Details at peptide level (e.g., sequence coverage)	+++	+
Number of modulated proteins identified	+	+++
Coupling with biochemical methods		
Antibodies	+++	+
Enzymes (zymography)	+	-
Robustness of quantification		
Sensitivity	++	+++
Linearity	+++	+
Need of validation	+++	+++

* Depending on 2-DGE technique used.

## Data Availability

Not applicable.
